# Nucleoplasmic checkpoint of the 40S ribosomal decoding center maturation

**DOI:** 10.1016/j.celrep.2026.117545

**Published:** 2026-06-11

**Authors:** Benjamin Lau, Yi Li, Jingyi Zhu, Xianwen Ye, Paulina Fischer, Xiaying Hong, Rui Yuan, Roland Beckmann, Ed Hurt, Jingdong Cheng

**Affiliations:** 1 Minhang Hospital & Institutes of Biomedical Sciences, Shanghai Key Laboratory of Medical Epigenetics, International Co-laboratory of Medical Epigenetics and Metabolism, Fudan University, Shanghai, China; 2 Heidelberg University Biochemistry Center (BZH), Heidelberg, Germany; 3 Gene Center and Department of Biochemistry, University of Munich LMU, Munich, Germany; 4 Molecular Systems Biology Unit, European Molecular Biology Laboratory (EMBL), Heidelberg, Germany

**Keywords:** rrp12, decoding center, quality control, ribosome assembly, 90S, pre-40S, premature RNA folding, *Chaetomium thermophilum*, helix44, helix28

## Abstract

The decoding center (DC) is a key ribosomal structure for accurate translation, assembled in a multi-step process that starts on nucleolar pre-ribosomes and ends in the cytoplasm. While late cytoplasmic steps and their checkpoint mechanisms are well characterized, the regulation of early nucleoplasmic DC assembly is unclear. Here, we show that the essential assembly factor Rrp12 plays a central coordinating role. Using *Chaetomium thermophilum* and cryo-electron microscopy analyses of fifteen pre-40S intermediates, we demonstrate that Rrp12 C terminus truncation: (1) inhibits release of the Utp14-Dhr1 pair, (2) displaces Tsr1, (3) promotes premature stabilization of h28, and (4) prevents h44 formation. These defects impair final 18S rRNA processing and prematurely activate the quality control kinase Rio1. Our results reveal a nucleoplasmic checkpoint during DC formation and establish Rrp12 as a critical regulator ensuring accurate assembly and orderly ribosome maturation.

## Highlights

• Rrp12 coordinates ordered decoding center assembly during nucleoplasmic 40S maturation
• Truncation of the Rrp12 C terminus causes premature h28 folding and loss of h44
• Misassembled nucleoplasmic pre-40S particles recruit Rio1 for nuclear quality control

## Graphical abstract

**Figure:**
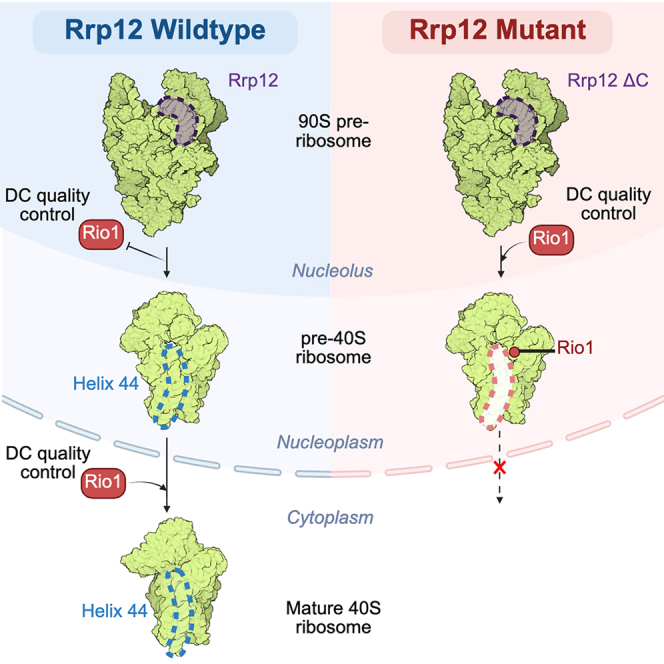

## Teaser

Lau et al. report that Rrp12 coordinates ordered decoding center assembly during 40S biogenesis by promoting timely assembly factor release and preventing premature rRNA folding. Rrp12 C-terminal truncation disrupts decoding center maturation, triggering premature recruitment of the Rio1 kinase, which could be a nucleoplasmic checkpoint to monitor 40S assembly fidelity.

## Introduction

Ribosomes are large ribonucleoprotein (RNP) complexes consisting of a small (40S) and large (60S) subunit, which together catalyze protein synthesis by decoding messenger RNAs (mRNAs). In eukaryotic cells, ribosome assembly begins in the nucleolus with the transcription of precursor ribosomal RNA (35S pre-rRNA in yeast) that contains the small (18S rRNA) and large subunit rRNAs (5.8S and 25S rRNA). Specific assembly factors (AFs) facilitate and guide its co-transcriptional folding and association with early ribosomal proteins.^1^^,^^2^^,^^3^^,^^4^^,^^5^ The biogenesis process of both ribosomal subunits follows a highly coordinated series of assembly, modification, and maturation steps, as the pre-ribosomes progress from the nucleolus into the nucleoplasm, concluding with the final maturation steps in the cytoplasm.^1^^,^^2^^,^^3^^,^^4^^,^^5^

Formation of the small subunit (SSU) involves extensive remodeling of the 18S rRNA and its associated proteins.^1^^,^^2^^,^^3^^,^^4^^,^^5^ Nucleolar biogenesis events lead to the formation of the 90S pre-ribosome (or SSU processome), the precursor of the 40S subunit, and the first biochemically stable pre-ribosomal particle. It is assembled co-transcriptionally on the 5′ external transcribed spacer (5′ ETS)^6^^,^^7^^,^^8^^,^^9^^,^^10^ and consists of early acting AFs and ribosomal proteins. Many of the factors within this particle are organized into modular subcomplexes, including UTP-A, UTP-B, and the U3 snoRNP, which together scaffold this nascent particle.^1^^,^^2^^,^^3^^,^^4^^,^^5^ As transcription progresses into the 18S rRNA region, additional AFs (e.g., Utp20, Rrp5, and Krr1), higher-order modules (e.g., Noc4 module, UTP-C, and Kre33 module), and snoRNAs (snR30 and U14) are incorporated to complete 90S pre-ribosome formation.^11^^,^^12^^,^^13^^,^^14^ Next, the transition from the huge 90S particle to the much smaller pre-40S ribosome marks a major structural and regulatory pivot in 40S biogenesis.^15^^,^^16^ It is initiated by endonucleolytic cleavage of 5′ ETS at site A_1_, which is followed by a gradual shedding of 90S AFs. Finally, nucleolar factors such as U3 snoRNA, Utp14, and the RNA helicase Dhr1 are released after Dhr1-mediated unwinding of the U3-18S duplex. This permits formation of the central pseudoknot, ultimately driving the compaction of the 18S rRNA core and establishing the first pre-40S intermediates.^15^^,^^17^^,^^18^^,^^19^^,^^20^ Concomitantly, nucleoplasmic factors including Enp1, Dim1, Ltv1, and Rrp12 assemble around the developing head and platform domains, guiding subsequent maturation steps that lead toward early decoding center (DC) formation and cytoplasmic export.^19^^,^^20^^,^^21^^,^^22^^,^^23^^,^^24^

The DC constitutes the functional core of the ribosome, ensuring the accuracy of mRNA decoding during translation.^25^^,^^26^ It undergoes particularly intricate remodeling during maturation, which begins in the nucleolus and is only completed after export to the cytoplasm.^19^^,^^20^^,^^21^^,^^22^ It involves ordered remodeling of key structural elements of the 18S rRNA, most notably helices 28 (h28) and 44 (h44). The late cytoplasmic stages of this pathway are well characterized, including the contributions of the methyltransferase Bud23, the methyltransferase Dim1, the endonuclease Nob1, the AF eIF1AD, and the quality control kinases Rio1 and Rio2.^19^^,^^20^^,^^21^^,^^22^^,^^27^^,^^28^^,^^29^^,^^30^^,^^31^^,^^32^^,^^33^ However, the molecular mechanisms behind the earlier nucleoplasmic DC formation events remain largely unknown.

Rrp12 is a highly conserved AF with well documented roles in multiple steps of 40S subunit biogenesis.^19^^,^^21^^,^^34^ It is first recruited to nucleolar 90S particles and remains associated with cytoplasmic pre-40S intermediates. While earlier studies implicated Rrp12 primarily in the nuclear export of pre-40S particles,^34^^,^^35^ more recent cryo-electron microscopy (cryo-EM) structures in human cells have revealed an additional role,^19^ indicating that Rrp12 acts as a central organizer of AFs during the formation and stabilization of the pre-40S head domain. Moreover, the C-terminal domain is positioned in proximity to the DC, suggesting that it may contribute to early nucleoplasmic remodeling events.^19^ Despite these discoveries, the exact mechanistic role of Rrp12 during DC formation remains unclear.

In this study, we used high-resolution cryo-EM in the thermophilic fungus *Chaetomium thermophilum* to map the structural changes of the DC during early 40S maturation. This organism, which preserves the eukaryotic ribosome assembly pathway with enhanced biochemical stability, provides a powerful model for visualizing transient intermediates.^9^^,^^11^ We determined fifteen cryo-EM structures of 40S assembly intermediates, spanning nucleolar 90S pre-ribosomes to cytoplasmic pre-40S particles, in both wild-type Rrp12 and a Rrp12 C-terminal truncation (ΔC) mutant. These structures show that Rrp12 plays a key role in coordinating the step-by-step folding of 18S rRNA h28 and 44, promoting the release of early AFs (e.g., Utp14 and Dhr1) and therefore, aiding the recruitment of late-acting factors (e.g., Bud23 and Rio1), and thus ensuring correct 3′ end processing of 18S rRNA. Truncating the Rrp12 C-terminal domain perturbs these mechanisms, which causes defects in 18S rRNA processing and triggers untimely surveillance activation by Rio1. Overall, these results establish Rrp12 as a critical regulator of DC remodeling during nucleoplasmic maturation and demonstrate its role in coupling structural transitions to downstream quality control pathways.

## Results

### Rrp12 C-terminal truncation mutants exhibit early structural maturation defects of the 40S decoding center

The DC of the eukaryotic 40S ribosome is a critical functional site that undergoes a complex maturation process during 40S subunit biogenesis. Despite the wealth of structural information describing the late stages of DC maturation in the cytoplasm,^19^^,^^20^^,^^21^^,^^22^^,^^23^^,^^24^^,^^36^^,^^37^^,^^38^ the structural basis underlying the early steps of DC folding in the nucleoplasm remains poorly understood. To bridge this gap, we sought to investigate the specific function of Rrp12 as the C terminus of the human RRP12 is positioned right at the DC and directly interacts with the BUD23-TRMT112 (yeast Bud23-Trm112) methyltransferase complex,^19^ which is important for DC maturation during the nucleoplasmic phase.

**Figure 1:**
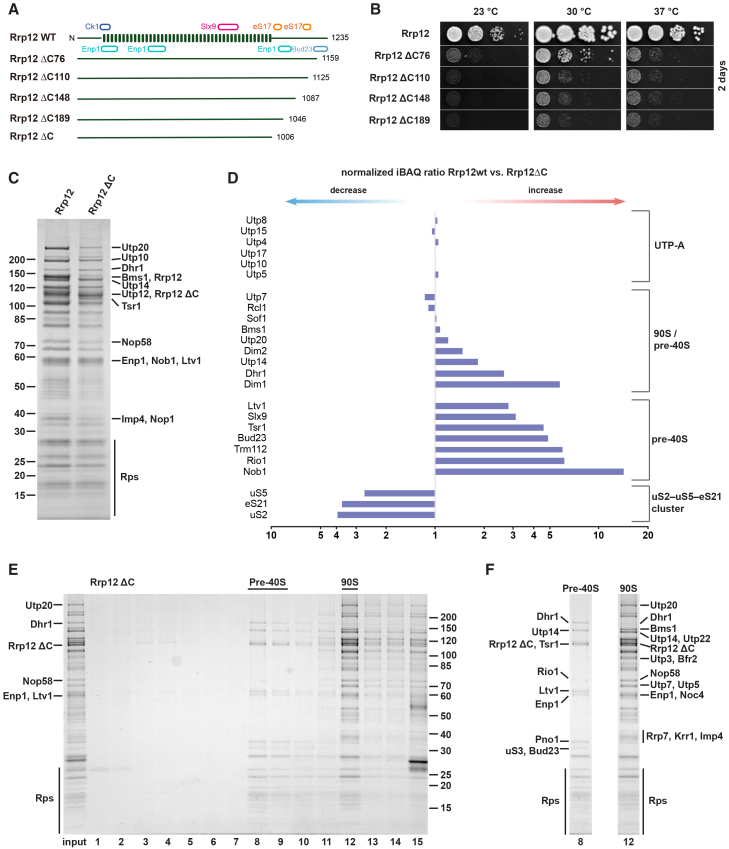
Rrp12 C-terminal truncations disrupt pre-ribosome assembly (A) Top: schematic representation of yeast Rrp12 protein architecture, highlighting the predicted interaction interfaces with indicated assembly factors. Bottom: schematic representations of various Rrp12 truncation mutants. (B) Dot-spot growth assay of wild-type *S. cerevisiae* (W303) and the indicated Rrp12 C-terminal truncation mutants. Serial dilutions of each strain were spotted onto YPD (Yeast extract Peptone Dextrose) plates and incubated for 2 days at 23°C, 30°C, or 37°C. (C) SDS-PAGE analysis of the *C*. *thermophilum* pre-ribosomal particles purified using Enp1-Rrp12 (left) or Enp1-Rrp12 ΔC (right) split-tag. Co-purified ribosome assembly factors identified by mass spectrometry are indicated. (D) Semi-quantitative mass spectrometry (semiQ-MS) analysis of pre-ribosomes purified by sequential two-step affinity purification via tagged Enp1 and Rrp12 from wild-type and mutant cells. Intensity-based iBAQ values were normalized to the UTP-A subunit Utp10, and log_10_ ratios (wild-type/mutant) are plotted according to their fold change. Selected assembly factors are grouped by their occurrence in 90S or pre-40S particles and by their corresponding biogenesis modules, as indicated on the right. The source data of the label-free quantitative LC-MS/MS are provided in Table S1. (E and F) SDS-PAGE analysis of *C. thermophilum* pre-ribosomal particles purified via split-tagged Enp1-Rrp12 ΔC and separated by sucrose gradient centrifugation (E). Gradient fractions corresponding to pre-40S and 90S particles are indicated. Fractions 8 (pre-40S) and 12 (90S) are shown separately on the right, with bands identified by mass spectrometry (F).

To investigate whether the C-terminal region of Rrp12 may play a direct role in coordinating the early structural maturation of the DC, we generated a series of Rrp12 ΔC mutants (Figure 1A) in *S. cerevisiae*, where the unstructured C terminus separates the structured region from the HEAT-repeat domain of Rrp12. All truncation variants exhibited cold-sensitive growth defects (Figure 1B), indicating that the C-terminal region is essential for optimal cell growth and functionally important for ribosome biogenesis.

To further dissect the function of Rrp12’s C terminus, we performed split-tag tandem affinity purification using Enp1 as first and Rrp12 or Rrp12 ΔC as second bait proteins in *C. thermophilum,* which has been shown to be advantageous for cryo-EM structural analyses of macromolecular complexes.^9^^,^^11^^,^^39^^,^^40^^,^^41^ SDS-PAGE analysis confirmed successful purification of pre-ribosomal complexes via Enp1-Rrp12, revealing both 90S and pre-40S ribosome particles (Figure 1C), consistent with findings from yeast and human.^16^^,^^33^^,^^42^ Semi-quantitative mass spectrometry analysis revealed that the Rrp12 ΔC variant remains capable of associating with 90S/40S pre-ribosomal intermediates and does not arrest particles during 90S ribosome assembly (Table S1), in agreement with previous findings.^11^ Compared to particles associated with wild-type Rrp12, the Rrp12 ΔC sample strongly enriched pre-40S biogenesis factors. Interestingly, dual 90S/pre-40S factors Dhr1 and Utp14 were increased in mutant particles, while other 90S AFs remained relatively unchanged (Figure 1D), indicating a defect in the 90S to pre-40S transition during early 40S biogenesis. Moreover, ribosomal proteins eS21, uS5, and uS2 were specifically reduced in the Enp1-Rrp12 ΔC preparation, suggesting that they are less stably associated with the pre-ribosomes.

To further characterize how the Rrp12 ΔC mutant affects the composition of assembly intermediates, purified Enp1-Rrp12 ΔC complexes were separated by sucrose gradient centrifugation (Figure 1E). Notably, in addition to canonical pre-40S factors, the pre-40S fraction contained 90S AFs Dhr1 and Utp14, which are normally released during the 90S to 40S transition in the nucleoplasm.^15^^,^^17^^,^^18^^,^^20^ Additionally, we found Rio1, a kinase typically associated with very late cytoplasmic pre-40S particles (Figure 1F).^21^^,^^22^^,^^30^ Together, our biochemical data suggest that the C terminus of Rrp12 is involved in a distinct pre-40S maturation event. Moreover, the abnormal factor retention indicates a disruption in the handoff between early nucleoplasmic and late cytoplasmic events during 40S ribosome maturation.

### Cryo-EM structures of thermophile Enp1-Rrp12 WT particles reveal conserved maturation route of the decoding center

**Figure 2:**
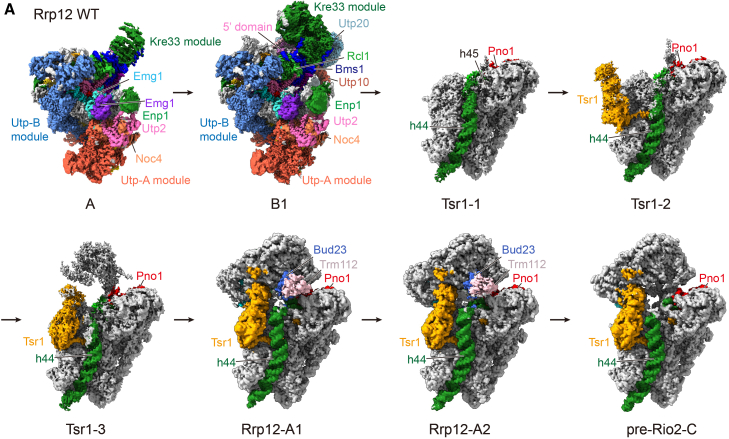
Cryo-EM structures of thermophile Enp1-Rrp12 WT particles (A) Cryo-EM analysis of *C. thermophilum* Enp1-Rrp12 WT complexes revealed eight distinct pre-ribosomal assembly states, including two 90S intermediates (states A and B1) and six sequential pre-40S intermediates (Tsr1-1, Tsr1-2, Tsr1-3, Rrp12-A1, Rrp12-A2, and pre-Rio2-C). Major assembly modules and factors are labeled in each map. All cryo-EM density maps shown were post-processed using DeepEMhancer only for visualization. See also Figures S1 and S3–S5 and Table S2.

Prompted by these biochemical findings, we performed single-particle cryo-EM analyses on pre-ribosomal particles purified from *C. thermophilum* expressing wild-type Rrp12. Using split-tag tandem affinity purification combined with extensive data processing, we resolved a total of eight distinct pre-ribosomal intermediates at molecular resolutions ranging from 2.9 to 3.3 Å (Figure 2; Figures S1–S5). These intermediates span almost the full range of 40S biogenesis, from early nucleolar to late cytoplasmic phases, including two 90S states (states A and B1, as published before^11^) and six pre-40S states representing progressive maturation stages (Tsr1-1, Tsr1-2, Tsr1-3, Rrp12-A1, Rrp12-A2, and pre-Rio2-C) (Figure 2). Structural models were obtained by combining *de novo* modeling with rigid-body fitting of AlphaFold-predicted structures^43^ (Table S2).

As previously characterized in yeast,^19^ the Tsr1 series (Tsr1-1 to Tsr1-3) represents early nucleoplasmic intermediates following the primordial pre-40S state Dis-C^15^ (Figure 2). These states are characterized by the sequential shedding of residual 90S-associated factors, coinciding with progressive stabilization and initial folding of the DC, specifically at h44 of the 18S rRNA. Subsequent nucleoplasmic states, Rrp12-A1 and Rrp12-A2, represent more advanced states competent for nuclear export, featuring substantial maturation of the 40S head, especially within the DC (Figure 2). In the Rrp12 states, a clear density was observed for the Bud23-Trm112 complex precisely positioned at the DC, consistent with previously reported yeast and human structures^19^ (Figure 2). However, the relatively weak density for Rrp12 and unresolved Slx9 density suggest substantial flexibility and dynamic interactions among ribosome AFs within these states (Figure S5).

The overall structural features of Rrp12-A1 and Rrp12-A2, including DC conformation, are strikingly similar to the previously characterized human counterparts (PDB: 7WTU and 7WTV, respectively).^19^ However, the late pre-Rio2-C state differed from previously characterized yeast and human structures^19^^,^^21^^,^^23^^,^^24^^,^^36^^,^^37^^,^^38^ by lacking detectable density for Rio2 (Figure 2, bottom right). Rio2 typically binds pre-40S particles during late cytoplasmic stages, mediating essential final maturation events at the DC.^19^^,^^21^^,^^23^^,^^24^^,^^36^^,^^37^^,^^38^ Therefore, the absence of clear Rio2 density in our pre-Rio2-C structure might reflect a transient and highly dynamic association, limiting its stable structural visualization. Alternatively, this feature could indicate a species-specific maturation pathway unique to *C. thermophilum.* Collectively, these results emphasize the highly conserved structural route underlying DC maturation, delineating key nucleoplasmic and cytoplasmic pre-40S intermediates.

### Cryo-EM structures of Enp1-Rrp12 ΔC particles reveal aberrant 40S maturation

**Figure 3:**
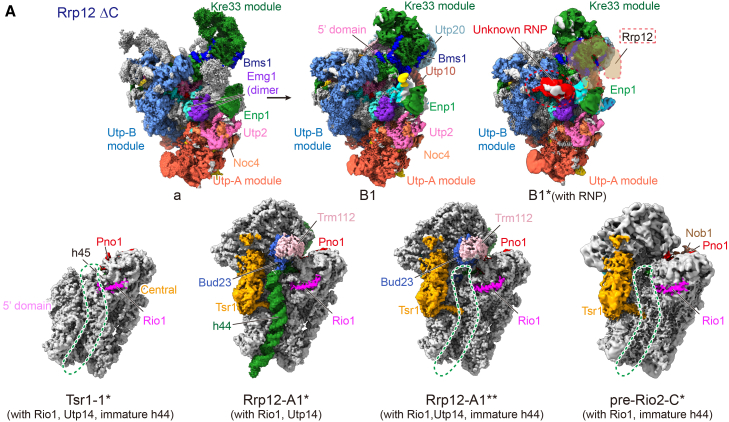
Cryo-EM structures of thermophile Enp1-Rrp12 ΔC particles (A) Cryo-EM analysis of *C. thermophilum* Enp1-Rrp12 ΔC complexes revealed three 90S states (a, B1, and B1^∗^) and four aberrant pre-40S intermediates (Tsr1-1^∗^, Rrp12-A1^∗^, Rrp12-A1^∗∗^, and pre-Rio2-C^∗^). The B1^∗^ state contains an unassigned protein/RNA density (highlighted in red) near Emg1, and the approximate position of the low resolution Rrp12 is indicated by a red dashed outline. The aberrant pre-40S particles are characterized by retention of Utp14, early recruitment of Rio1, as well as the immature h44. Position for the missing h44 is marked by green dashed lines. See also Figures S2–S4, S6, and S7; Table S3.

To further elucidate the specific role of the Rrp12 C-terminal region in ribosome biogenesis, we performed single-particle cryo-EM on pre-ribosomal particles purified from *C. thermophilum* expressing the Rrp12 ΔC mutant (Figure 3; Figures S2–S4, and S6; Table S3). In addition to capturing canonical 90S states a and B1, we identified another 90S state, which we term B1^∗^ (Figure 3, top right). This state closely resembles B1 in its overall architecture, but it contains an additional density near Emg1 and Rrp12, a region that is empty in the state B1 map (Figure 3; Figures S7A and S7B). This extra density displays features consistent with those of both protein and RNA (Figure S7C), suggesting that it may represent an RNP complex, possibly a snoRNP involved in rRNA modification within the 3′ major domain. Although the resolution of this density is insufficient to define its precise identity, its close spatial association with Rrp12 raises the possibility that Rrp12 may transiently coordinate with rRNA modifying enzymes during early assembly. Loss of the Rrp12 C terminus may impair this coordination, leading to the retention of the putative RNP on the 90S pre-ribosome.

Despite this retention, 90S pre-ribosomes in the Rrp12 ΔC mutant still efficiently transition into pre-40S particles. Particle classification revealed a comparable distribution of 90S intermediates between wild-type and mutant samples (Figure S4A), indicating that early assembly is not severely affected. In contrast, a strong redistribution is observed within the pre-40S population, with marked enrichment of Rrp12-associated intermediates in the mutant and a concomitant reduction of both upstream Tsr1 and downstream Rio2 states relative to the wild type. This indicates a bottleneck at the Rrp12 state of pre-40S maturation, impairing progression toward later intermediates. In the pre-40S population, we identified four structurally abnormal intermediates that we designated Tsr1-1^∗^, Rrp12-A1^∗^, Rrp12-A1^∗∗^, and pre-Rio2-C^∗^ (Figure 3; Figure S6). These states differ markedly from their wild-type intermediates both in composition and structural maturation. Notably, three of these intermediates (except state Rrp12-A1^∗^) lack visible density for h44, suggesting disrupted DC formation during the maturation (Figure 3). All identified Rrp12 ΔC pre-40S particles prematurely recruited Rio1, indicating early activation of a surveillance mechanism^31^^,^^38^^,^^44^ (Figure 3). Additionally, except for the pre-Rio2-C^∗^ state, these pre-ribosomes persistently retained Utp14 (Figure 3; Figure S6), a 90S AF known to activate the RNA helicase Dhr1 during the transition from 90S to pre-40S particles.^17^ While a Dhr1 density could not be directly seen in our cryo-EM maps, its known stable association with Utp14 suggests that it may be present in a flexible unresolved conformation.

Together, the deletion of the Rrp12 C terminus disrupts the orderly release of early pre-40S AFs and the recruitment of late maturation factors. While Rrp12 ΔC remains capable of assembling into 90S particles, the persistent presence of Utp14 and Rio1 in downstream intermediates indicates a failure in the transition during nucleoplasmic to cytoplasmic maturation. This defect could cause the cold-sensitive growth phenotype observed in the yeast *rrp12 ΔC* strains (see Figure 1B) and supports a model, in which the Rrp12 C terminus facilitates DC maturation by guiding timely AF release and recruitment.

### Persistent Utp14 binding in Rrp12 ΔC pre-40S intermediates blocks 3′ end maturation of 18S rRNA

During the final cytoplasmic maturation of the 40S ribosomal subunit, the endonuclease Nob1 cleaves a short fragment from the 3′ end of the 18S rRNA to generate the mature 40S ribosome.^22^^,^^29^ Nob1 is recruited to pre-40S particles via two key interaction interfaces: one with Pno1 and another directly shielding the 3′ end of the 18S rRNA.^21^ In the primordial state Dis-C, both Nob1-binding sites are occupied by Utp14, which acts in concert with the RNA helicase Dhr1 to promote pre-rRNA remodeling.^15^

**Figure 4:**
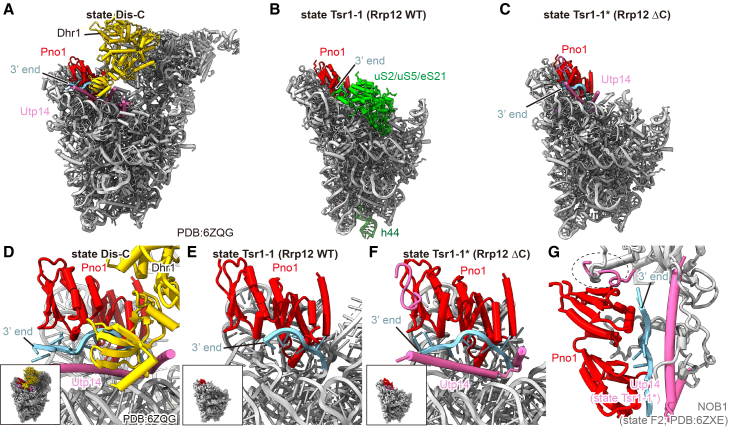
Retention of Utp14 prevents the cleavage of the 3′ end of 18S rRNA (A–C) Cryo-EM structures of 40S pre-ribosome near the 3′ end of 18S rRNA: Dis-C (PDB: 6ZQG, A), Tsr1-1 from Rrp12 WT (B), and Tsr1-1^∗^ from Rrp12 ΔC (C). Relevant assembly factors near the 3′ end are highlighted. In Tsr1-1^∗^, Utp14 remains associated with the particle. (D–F) Close-up views on the interface of Pno1 and Utp14 near the 3′ end of 18S rRNA in Dis-C (D), Tsr1-1 (E), and Tsr1-1^∗^ (F). (G) Superposition of Utp14 in state Tsr1-1^∗^ and NOB1 in human state F2 (PDB: 6ZXE) show steric clash between Utp14 and NOB1.

Under normal biogenesis conditions, the release of Utp14 and Dhr1 during the Dis-C to Tsr1-1 transition (Figures 4A–4E) exposes these binding sites,^20^ allowing Nob1 to dock correctly and execute the final rRNA processing. However, in the states Tsr1-1^∗^, Rrp12-A1^∗^, and Rrp12-A1^∗∗^, Utp14 remains bound to Pno1 and the 3′ end of the 18S rRNA (Figures 4C and 4F; Figures S4B and S6), directly occupying the sites required for Nob1 engagement. This retention creates a steric clash between Utp14 and Nob1 (Figure 4G), thereby preventing proper cleavage of the rRNA and consequently inhibiting the final pre-40S maturation step. We conclude that the C-terminal truncations of Rrp12 interfere with the release of early nucleoplasmic factors, resulting in sterical clashes that prevent further progression of pre-40S biogenesis.

### Premature recruitment of Rio1 in Rrp12 ΔC mutant marks defective pre-40S particles for surveillance

Rio1 functions as a late-acting surveillance kinase that monitors the structural integrity of the DC and ensures accurate 3′ end cleavage of the 18S rRNA.^22^^,^^30^^,^^31^^,^^44^ Recent studies have shown that Rio1 preferentially associates with correctly cleaved rRNA and serves as a proofreading checkpoint to prevent misprocessed or structurally defective pre-40S particles from entering the translating pool.^31^^,^^44^

**Figure 5:**
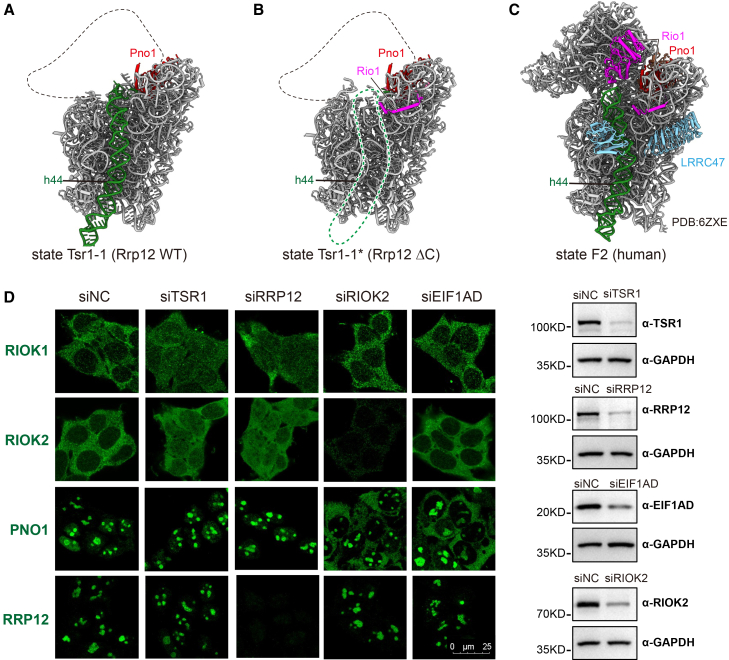
Early recruitment of Rio1 to nucleoplasmic pre-40S ribosomes in Rrp12 ΔC sample (A–C) Cryo-EM structures of the pre-40S state Tsr1-1 from Rrp12 WT (A), Tsr1-1^∗^ from Rrp12 ΔC (B), and the cytoplasmic human pre-40S state F2 (PDB: 6ZXE, C). In the Rrp12 ΔC mutant, Rio1 is aberrantly recruited at early nuclear states (Tsr1-1^∗^), in contrast to its expected cytoplasmic association. The flexible head domain of the pre-40S particle in the Rrp12 ΔC state was poorly resolved and is indicated by a dashed outline. Key structural elements, including Rio1, Pno1, LRRC47, and h44 of 18S rRNA, are color-highlighted. (D) Immunofluorescence analysis of the subcellular distribution of endogenous RIOK1, RIOK2, PNO1, and RRP12 in HEK293T cells following knockdown of TSR1, RRP12, RIOK2, or EIF1AD, as indicated. Knockdown efficiency of each target protein was confirmed by immunoblotting (right). Scale bars, 25 μm.

We observed that in wild-type pre-40S intermediates, such as the Tsr1-1 state, Rio1 is absent and h44 is properly formed, reflecting a normal nucleolar to nucleoplasm transition (Figures 2 and 5A). In contrast, in Rrp12 ΔC mutant pre-ribosomes, Rio1 is already bound to the pre-40S states within the nucleoplasm, such as the earliest Tsr1-1^∗^ state (Figures 3 and 5B; Figure S4C). The C-terminal region of Rio1 is positioned near the DC, similarly to its conformation in the human F2 state (PDB: 6ZXE),^22^ despite the improperly folded DC (Figures 3 and 5C).

To complement our structural analysis, we analyzed the subcellular localization of RIOK1/Rio1 and RRP12 in human cells using immunofluorescence (IF) under siRNA-mediated knockdown conditions. Under steady-state conditions, RRP12 is predominantly nuclear, consistent with its role in early ribosome biogenesis. Upon depletion of RRP12 or TSR1, RIOK1/Rio1 shows a marked increase in nuclear localization compared with control cells, indicating a shift in its subcellular distribution when early pre-40S ribosome biogenesis is impaired and supporting premature nucleoplasmic engagement of RIOK1/Rio1 (Figure 5D). This premature recruitment suggests that Rio1 engages misfolded intermediates and may act as a quality control sensor.

### Untimely folding of h28 impairs decoding center maturation in Rrp12 ΔC particles

**Figure 6:**
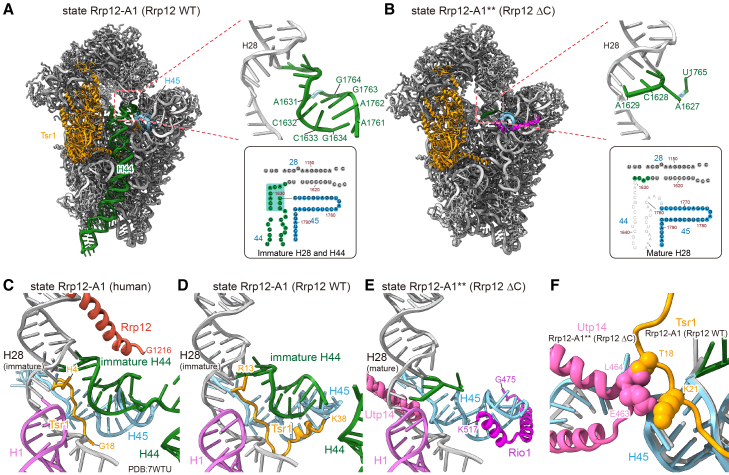
Precocious matured h28 prevents the maturation of DC (A and B) Left: molecular model of state Rrp12-A1 from Rrp12 WT sample and Rrp12-A1^∗∗^ from Rrp12 ΔC sample. Top right: close-up views of the RNA helices, showing immature h28/h44 in state Rrp12-A1 and precocious matured h28 in state Rrp12-A1^∗∗^. Bottom right: depiction of immature base pairing of h28 and h44 (Rrp12-A1) and precociously mature h28 (Rrp12-A1^∗∗^) with corresponding rRNA secondary structures. (C–E) Close-up views on the location of N terminus of Tsr1, Utp14, and Rio1 surrounding h28 and h44 in human state Rrp12-A1 (PDB: 7WTU, C), *C. thermophilum* Rrp12-A1 (D), and Rrp12-A1^∗∗^ (E). Functional elements are color-coded and labeled. (F) Superposition shows steric clash between Utp14 in state Rrp12-A1^∗∗^ and N terminus of Tsr1 in state Rrp12-A1.

To understand the structural basis for DC defects in the Rrp12 ΔC mutants, we analyzed the conformation of h28 and h44 across pre-40S intermediates. In wild-type particles such as Rrp12-A1, h28 and h44 adopt immature conformations stabilized by immature base pairs, consistent with the human pre-40S ribosome (PDB: 7WTU, Figures 6A–6D).^19^ This conformation is thought to facilitate the binding of key maturation factors including Bud23 and Rio2, which drive further maturation.^21^

In contrast, in the pre-40S states isolated via Rrp12 ΔC (such as state Rrp12-A1^∗∗^), h28 is already matured, forming right Watson-Crick base pairs (Figure 6B). Although this early h28 maturation does not preclude Bud23-Trm112 incorporation, it coincides with the loss of density for h44, suggesting that early folding of h28 disrupts the coordinated formation of the DC.

Mechanistically, our structural comparison highlights a critical role for the N-terminal region of Tsr1 (residues R13-K38), which normally inserts between h28 and h44 to sterically prevent untimely folding of h28, leading to the immature base pairing of h28 and h44 and support proper DC maturation (Figures 6C and 6D; Figure S4D).^19^ However, in the cryo-EM states derived from mutant particles, such as the Rrp12-A1^∗∗^ state, although Tsr1 remains bound, the N-terminal region is absent from the density map. Instead, this region is occupied by Rio1 and Utp14 (Figure 6E). Importantly, the side chains of Utp14 residues E463 and L464 directly clash with Tsr1 residues T18 and K21 (Figure 6F), explaining the displacement of Tsr1’s N terminus from the pre-40S.

Thus, the persistent retention of Utp14 in Rrp12 ΔC particles interferes with the correct positioning of the Tsr1 N terminus, allowing untimely maturation of h28 and destabilizing h44. This misfolded DC configuration could then be recognized by Rio1, which then may engage the particle as a part of a quality control checkpoint, marking the particle as defective and blocking its progression along the maturation pathway.

## Discussion

**Figure 7:**
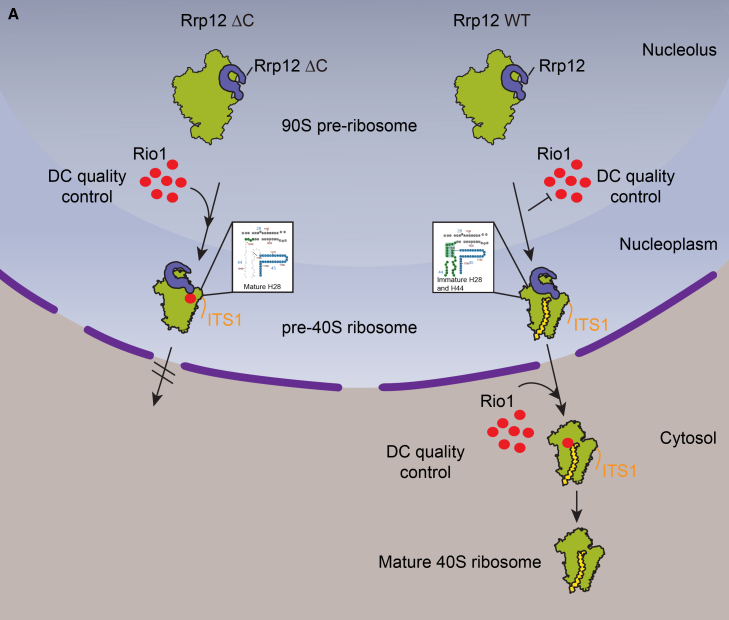
Rrp12 regulates decoding center maturation through a nucleoplasmic checkpoint (A) A proposed model illustrating the role of Rrp12 in the maturation of the decoding center. In wild-type cells (right), timely release of Utp14 and Dhr1 from the 90S pre-ribosome enables h28 and h44 maturation and proper formation of the decoding center. The resulting pre-40S particle passes the quality control step mediated by Rio1 and finally matures in the cytoplasm. In contrast, truncation of the Rrp12 C terminus (left) impairs Utp14-Dhr1 release, causing premature stabilization of h28, failure of h44 formation, and accumulation of immature pre-40S intermediates in the nucleus. These defective particles are recognized by the Rio1 quality control system, preventing their progression to functional ribosomes.

In this study, we observed that Rrp12 plays a key role in coordinating early steps of DC assembly and maintaining maturation fidelity during the transition from 90S to pre-40S ribosome (Figure 7). Our data suggest that the Rrp12 C-terminal region contributes to pre-40S maturation in a stepwise manner. Consistent with this idea, gradual truncation of the Rrp12 C terminus causes progressively stronger growth defects, indicating that this region is not required simply for a single assembly step. Instead, it may coordinate multiple events during early 40S biogenesis. One possible mechanism is that the Rrp12 C-terminal region coordinates with pre-40S AFs to help maintain h28 and the DC in an immature conformation. This could ensure that the DC remains one of the last regions to mature during 40S assembly. Loss of this region may, therefore, disrupt the ordered timing of DC maturation, explaining why the Rrp12 ΔC mutant shows premature h28 maturation and premature Rio1 recruitment, although the precise molecular sequence of events cannot be resolved from the present dataset.

While we cannot formally exclude that a subset of particles might represent dead-end intermediates, several observations suggest that the particles derived from the Rrp12 ΔC mutant correspond to on-pathway intermediates stalled at a defined checkpoint. First, except for the missing h44 in the 18S rRNA, these particles retain a largely intact pre-40S architecture, rather than showing the extensive fragmentation or collapse expected for nonspecific degradation products.^45^ Second, the observed states are not structurally random, but form a sequential maturation series that can be related to known pre-40S biogenesis intermediates.^19^^,^^21^ Their major defects are concentrated around the DC, h44, Utp14, Rio1, and Rrp12-dependent region, consistent with a specific delay in DC maturation rather than random defects. Third, the selective retention of early factors such as Utp14 and the premature recruitment of the surveillance factor Rio1 are consistent with a stalled assembly trajectory, and our IF data showing nucleoplasmic accumulation of Rio1 upon Rrp12 knockdown further indicate engagement of a Rio1-dependent nuclear quality control pathway rather than stochastic decay. Together, these observations lead us to suggest that these mutant particles represent stalled pre-40S biogenesis intermediates.

The structural core of the DC is formed by h44, and it is essential for codon-anticodon recognition.^25^^,^^26^ Its loss in multiple Rrp12 ΔC intermediates suggests that the timely and hierarchical RNA folding and factor exchange is disrupted. These structural defects are accompanied by premature recruitment of the quality control kinase Rio1, which is normally recruited during late cytoplasmic maturation. Recent works have demonstrated that Rio1 plays a quality control role in the cytosol by monitoring the accuracy of Nob1-mediated 18S rRNA 3′ end cleavage in yeast—nascent ribosomes with miscleaved rRNA accumulate and enter translating polysomes if the Rio1 checkpoint is bypassed.^31^^,^^44^ Our findings suggest that Rio1 acts not only as a terminal cytoplasmic checkpoint factor but can also serve as a surveillance sensor capable of recognizing misfolded nuclear intermediates.

The retention of Utp14 in the Rrp12 ΔC mutants provides mechanistic insight into this misregulation. In wild-type pre-40S particles, Utp14 is released early to allow proper positioning of Tsr1 and ordered folding of h28 and h44. In pre-ribosomes containing Rrp12 ΔC, Utp14 occupies the space normally reserved for Tsr1’s N-terminal region, displacing this critical regulator. Without Tsr1’s restraint, h28 prematurely adopts a fully mature conformation. This, in turn, destabilizes h44, likely by preventing the correct three-way junction formation required for its docking. This is in line with recent DMS-MaPseq analysis, which reveals that three-helix junctions tend to misfold during SSU assembly *in vivo*. Ribosome AFs help ensure correct RNA folding by preventing premature tertiary interactions that could become kinetically trapped, leading to arrested ribosome biogenesis.^46^ Interestingly, among the aberrant intermediates identified in the Rrp12 ΔC mutant, the Rrp12-A1^∗^ state uniquely retains a well-resolved h44, despite the early recruitment of Rio1 and retention of Utp14. This observation suggests that while truncation of Rrp12’s C terminus broadly disrupts the normal coordination of DC maturation, some pre-40S particles may still transiently achieve correct h44 folding.

Surprisingly, the premature maturation of h28 and concomitant destabilization of h44 in Rrp12 ΔC pre-40S particles show conceptual parallels to the bacterial 30S ribosomal subunit, where helix h28 can adopt an alternative hALT conformation that modulates the balance between inactive and active subunit states.^47^^,^^48^ In bacteria, this reflects a reversible conformational equilibrium that contributes to functional tuning of the ribosome. In contrast, in eukaryotic ribosome biogenesis, h28 folding appears to be tightly controlled by AF-dependent timing rather than by intrinsic RNA conformational equilibria. Thus, h28 acts as a structural regulator in both systems, but its functional role is governed by distinct regulatory principles in bacteria and eukaryotes.

In conclusion, our study revealed that Rrp12 is not merely a passive factor with scaffold properties but actively coordinates remodeling during the nucleoplasmic phase of DC assembly. Its C-terminal domain may function as a timing device by promoting steps, which lead to Utp14-Dhr1 release, stabilizing immature h28/h44 configuration and gating Rio1 engagement. Loss of Rrp12’s C terminus disrupts these transitions, leading to structural heterogeneity, assembly arrest, and quality control activation. Thus, our study provides a mechanistic framework for understanding how assembly fidelity is enforced during early ribosome maturation and sets the stage for identifying additional nucleoplasmic regulators.

### Limitations of the study

We identified a timely series of cryo-EM states along the biogenesis pathway of the 40S pre-ribosome, but we cannot rule out that we missed transient intermediates in our study. Furthermore, we note that cryo-EM provides static snapshots and does not directly establish the temporal order of the observed intermediates. Therefore, we cannot formally exclude the possibility that the identified states do not follow a strictly linear sequence. Nevertheless, their coherent structural and compositional differences are consistent with a progressive maturation pathway of the 40S subunit. For the Rrp12 ΔC, we did not observe an arrested 90S-to-pre-40S particle, which could explain the direct recruitment of the Rio1 quality control factor or why Utp14 and Dhr1 remain on the pre-40S ribosome; this might be due to transient intermediates. Although our structural and IF data support the interpretation that the Rrp12 ΔC particles represent stalled pre-40S particles, we cannot exclude that prolonged assembly arrest in the mutant background may generate minor off-pathway intermediates. Additionally, in this study, we did not analyze whether the Rrp12 ΔC mutant intermediate can be exported or remains in the nucleus.

## Resource availability

### Lead contact

Requests for further information and resources should be directed to and will be fulfilled by the lead contact, Dr. Jingdong Cheng (cheng@fudan.edu.cn).

### Materials availability

This study did not generate new unique reagents. Plasmids generated in this study are available from the lead contact without restriction.

### Data and code availability

• The authors declare that all the data supporting the findings of this study are available within the paper. All cryo-EM maps and molecular models have been deposited in the Electron Microscopy Data Bank (EMDB) and in the Protein DataBank (PDB) with accession codes: PDB: 9XA7 and EMD-66677 for 90S pre-ribosome (Enp1-Rrp12 WT) in state A; PDB: 9XA8 and EMD-66678 for 90S pre-ribosome (Enp1-Rrp12 WT) in state B1; PDB: 9XA9 and EMD-66679 for pre-40S ribosome (Enp1-Rrp12 WT) in state Tsr1-1; PDB: 9XAA and EMD-66680 for pre-40S ribosome (Enp1-Rrp12 WT) in state Tsr1-2; PDB: 9XAB and EMD-66681 for pre-40S ribosome (Enp1-Rrp12 WT) in state Tsr1-3; PDB: 9XAC and EMD-66682 for pre-40S ribosome (Enp1-Rrp12 WT) in state Rrp12-A1; PDB: 9XAD and EMD-66683 for pre-40S ribosome (Enp1-Rrp12 WT) in state Rrp12-A2; PDB: 9XAE and EMD-66684 for pre-40S ribosome (Enp1-Rrp12 WT) in state pre-Rio2-C; PDB: 9XAF and EMD-66685 for 90S pre-ribosome (Enp1-Rrp12 ΔC) in state a; PDB: 9XAG and EMD-66686 for 90S pre-ribosome (Enp1-Rrp12 ΔC) in state B1; PDB: 9XAH and EMD-66687 for 90S pre-ribosome (Enp1-Rrp12 ΔC) in state B1^∗^; PDB: 9XAI and EMD-66688 for pre-40S ribosome (Enp1-Rrp12 ΔC) in state Tsr1-1^∗^; PDB: 9XAJ and EMD-66689 for pre-40S ribosome (Enp1-Rrp12 ΔC) in state Rrp12-A1^∗^; PDB: 9XAK and EMD-66690 for pre-40S ribosome (Enp1-Rrp12 ΔC) in state Rrp12-A1^∗∗^; and EMD-66691 for pre-40S ribosome (Enp1-Rrp12 ΔC) in state pre-Rio2-C^∗^.
• Original western blot images have been deposited at Mendeley at https://doi.org/10.17632/nvr5z4g943.1 and are publicly available as of the date of publication. Microscopy data reported in this paper will be shared by the lead contact upon request.
• This paper does not report original code.
• Any additional information required to reanalyze the data reported in this paper is available from the lead contact upon request.

## Acknowledgments

We thank the Center of Cryo-Electron Microscopy, Core Facility of Shanghai Medical College at the Fudan University for technical support. This research was supported by grants from the 10.13039/501100012166National Key R&D Program of China (2025YFA1308703), the 10.13039/501100001809National Natural Science Foundation of China (32371350 and 32571495), and Fudan University and Cao’ejiang Basic Research (24FCB02) to J.C. and an ERC grant ADG 741781 GLOWSOME to E.H.

## Author contributions

Conceptualization, E.H. and J.C.; methodology, B.L., Y.L., J.Z., X.Y., P.F., X.H., and R.Y.; investigation, B.L., Y.L., J.Z., X.Y., P.F., X.H., and R.Y.; writing – original draft, B.L., Y.L., J.Z., X.Y., P.F., E.H., and J.C.; writing – review and editing, B.L., R.B., E.H., and J.C.; funding acquisition, E.H. and J.C.; supervision, E.H. and J.C.

## Declaration of interests

The authors declare no competing interests.

## STAR★Methods

### Key resources table

**Table:** 

REAGENT or RESOURCE	SOURCE	IDENTIFIER
**Antibodies**		

Anti-FLAG	SIGMA-Aldrich	A8592; RRID: AB_439702
Anti-ProteinA	SIGMA-Aldrich	P1291; RRID: AB_260996
Anti-RIOK1	Proteintech	17222-1-AP; RRID: AB_2284990
Anti-RIOK2	ABclonal	A12122; RRID: AB_2759012
Anti-RRP12	Proteintech	26849-1-AP; RRID: AB_3669571
Anti-TSR1	ABclonal	A4842; RRID: AB_2765899
Anti-EIF1AD	Proteintech	20528-1-AP; RRID: AB_10693533
Anti-PNO1	ABclonal	A17736; RRID: AB_2771729

**Bacterial and virus strains**		

*Escherichia coli* DH5α	Thermo Fisher Scientific	N/A

**Chemicals, peptides, and recombinant proteins**		

FLAG Peptide	CASLO	N/A
TEV protease	This study	N/A
SIGMA*FAST*	SIGMA-Aldrich	S8830
RiboLock RNase inhibitor	Thermo Scientific	EO0381

**Critical commercial assays**		

ANTI-FlagM2 Affinity Gel	SIGMA-Aldrich	A2220
IgG–Sepharose 6 Fast Flow	GE Healthcare	17096902

**Deposited data**		

Enp1-Rrp12 WT state A	This study	PDB: 9XA7, EMD-66677
Enp1-Rrp12 WT state B1	This study	PDB: 9XA8, EMD-66678
Enp1-Rrp12 WT state Tsr1-1	This study	PDB: 9XA9, EMD-66679
Enp1-Rrp12 WT state Tsr1-2	This study	PDB: 9XAA, EMD-66680
Enp1-Rrp12 WT state Tsr1-3	This study	PDB: 9XAB, EMD-66681
Enp1-Rrp12 WT state Rrp12-A1	This study	PDB: 9XAC, EMD-66682
Enp1-Rrp12 WT state Rrp12-A2	This study	PDB: 9XAD, EMD-66683
Enp1-Rrp12 WT state pre-Rio2-C	This study	PDB: 9XAE, EMD-66684
Enp1-Rrp12 ΔC state a	This study	PDB: 9XAF, EMD-66685
Enp1-Rrp12 ΔC state B1	This study	PDB: 9XAG, EMD-66686
Enp1-Rrp12 ΔC state B1^∗^	This study	PDB: 9XAH, EMD-66687
Enp1-Rrp12 ΔC state Tsr1-1^∗^	This study	PDB: 9XAI, EMD-66688
Enp1-Rrp12 ΔC state Rrp12-A1^∗^	This study	PDB: 9XAJ, EMD-66689
Enp1-Rrp12 ΔC state Rrp12-A1^∗∗^	This study	PDB: 9XAK, EMD-66690
Enp1-Rrp12 ΔC state pre-Rio2-C^∗^	This study	EMD-66691
Raw western blot	This study	Mendeley Data: https://doi.org/10.17632/nvr5z4g943.1

**Experimental models: Cell lines**		

Human: HEK293T	ATCC	CRL-3216

**Experimental models: Organisms/strains**		

*Chaetomium thermophilum* wild-type	DMSZ, Braunschweig	DSM 1495 https://www.dsmz.de
P_*ACT1*_-*HPHNT1*-T_*GPD*_-P_*ENP1*_-*ENP1*-TEV-ProtA-T_*GPD*_, P_*ACT1*_-*ERG1*-T_*GPD*_-P_*RRP12*_-*RRP12*-Flag-T_*GPD*_	This study	CT120
P_*ACT1*_-*HPHNT1*-T_*GPD*_-P_*ENP1*_-*ENP1*-TEV-ProtA-T_*GPD*_, P_*ACT1*_-*ERG1*-T_*GPD*_-P_*RRP12*_-*RRP12ΔC* -Flag-T_*GPD*_	This study	CT121
*ade2-1,trp1-1, leu2-3,112, his3-11,15, ura3-1, can1-100*	Thomas et al.^49^	W303
W303, *rrp12*::*HIS3*, [pRS316 *RRP12*]	Cheng et al.^11^	Rrp12 shuffle

**Oligonucleotides**		

siTSR1: GGCUGCUCGAAUUCGAUUUTT	This study	N/A
siRRP12: CCAGUGAGAAUGAUUUACATT	This study	N/A
siEIF1AD: CGCAGACAGUAUCAUGAGATT	Ameismeier et al.^22^	N/A
siRIOK2: GCCUUGUCGUCAUUAAAUATT	This study	N/A

**Recombinant DNA**		

p*HPH*_P_*ENP1*_-*ENP1*-TEV-ProtA-T_*GPD*_	This study	pBL002
p*ERG1*_P_*RRP12*_-*RRP12*-Flag-T_*GPD*_	This study	pBL248
p*ERG1*_P_*RRP12*_-*RRP12ΔC* -Flag-T_*GPD*_	This study	pBL249
P_RRP12_-*RRP12*-T_ADH1_, *URA3*, ARS/CEN, *AmpR*	Cheng et al.^11^	pRS316 Rrp12 WT
P_RRP12_-*RRP12*-T_ADH1_, *TRP1*, ARS/CEN, *AmpR*	Cheng et al.^11^	pRS314 Rrp12 WT
P_RRP12_-*RRP12*-T_ADH1_, *TRP1*, ARS/CEN, *AmpR*	This study	pRS314 Rrp12ΔC76
P_RRP12_-*RRP12*-T_ADH1_, *TRP1*, ARS/CEN, *AmpR*	This study	pRS314 Rrp12ΔC110
P_RRP12_-*RRP12*-T_ADH1_, *TRP1*, ARS/CEN, *AmpR*	This study	pRS314 Rrp12ΔC148
P_RRP12_-*RRP12*-T_ADH1_, *TRP1*, ARS/CEN, *AmpR*	This study	pRS314 Rrp12ΔC189
P_RRP12_-*RRP12*-T_ADH1_, *TRP1*, ARS/CEN, *AmpR*	Cheng et al.^11^	pRS314 Rrp12ΔC

**Software and algorithms**		

USCF ChimeraX	Goddard et al.^50^	http://www.cgl.ucsf.edu/chimerax
EPU 2	Thermo Fisher Scientific	https://www.thermofisher.cn/cn/zh/home/electron-microscopy/products/software-em-3d-vis/epu-software.html
Gautomatch	Kai Zhang	https://github.com/JackZhang-Lab/Gautmatch/tree/main/bin
DeepEMhancer	Sanchez-Garcia er al.^51^	https://github.com/rsanchezgarc/deepEMhancer
Coot	Emsley et al.^52^	https://www2.mrc-lmb.cam.ac.uk/personal/pemsley/coot/
MaxQUANT	Cox et al.^53^	https://www.maxquant.org
MotionCor2	Zheng et al.^54^	https://emcore.ucsf.edu/cryoem-software
CTFFIND4	Rohou et al.^55^	http://grigoriefflab.janelia.org/ctffind4
CryoSPARC	Punjani et al.^56^	https://structura.bio
Relion	Zivanov et al.^57^	https://www3.mrc-lmb.cam.ac.uk/relion/index.php/Main_Page
PHENIX	Adams et al.^58^	https://www.phenix-online.org
Alphafold	Jumper et al.^59^	https://alphafold.com

**Other**		

Cu300 R1.2/1.3 Grids	Quantifoil Micro Tools GmbH	N/A

### Experimental model and study participant details

#### Bacterial strains

For plasmid construction, the *E. coli* DH5α (Thermo Fisher Scientific) strain was used.

#### *Chaetomium thermophilum* strains

Used *Chaetomium thermophilum* strains and their genotypes are listed in the key resources table. *C. thermophilum* strains used in this study were derived from the DSM 1495 wild-type strain (DSMZ, Braunschweig, Germany). Epitope-tagged strains were generated as described previously.^60^ For split-tag tandem affinity purification, double-tagged strains were constructed by sequential transformation of *C. thermophilum* with two C-terminally tagged constructs: one encoding Enp1-ProtA as first bait and the other encoding either Rrp12-Flag or Rrp12 ΔC-Flag as the secondary bait, following established procedures.^11^

#### Yeast strains

The strains of *Saccharomyces cerevisiae* used in this study are listed in key resources table. Truncation variants were generated as described previously.^11^ All strains are derived from W303, *Saccharomyces cerevisiae* W303 strains were cultured in YPD medium at 30°C unless otherwise indicated.

#### Human cell lines

Human HEK293T cells were cultured in Dulbecco’s Modified Eagle Medium (DMEM) (BasalMedia Technologies, China) supplemented with 10% fetal bovine serum (FBS) (Excell Bio) and 1% penicillin/streptomycin (BasalMedia Technologies, China). Cell line identity was not authenticated by the authors, but cells were tested negative for mycoplasma contamination.

### Method details

#### Split-tag tandem affinity purification

*C. thermophilum* mycelium was harvested after 20 h at 50°C, washed, vacuum-dried, and frozen in liquid nitrogen. Frozen mycelium was disrupted using a cryogenic mill (Retsch MM400) in lysis buffer [60 mM Tris-HCl, pH 8.0, 40 mM KCl, 50 mM NaCl, 2 mM MgCl_2_, 5% glycerol, 1 mM DTT, 0.1% NP-40, EDTA-free protease inhibitors (SIGMAFAST), 0.013 U/μL RiboLock RNase Inhibitors (Thermo Scientific)]. Lysates were cleared by sequential centrifugation (10 min at 4,600 × g, 4°C; 20 min at 35,000 × g, 4°C) and incubated with IgG Sepharose 6 Fast Flow beads (GE Healthcare) for 12 h at 4°C. Beads were washed with 20 mL buffer (60 mM Tris-HCl, pH 8.0, 40 mM KCl, 15 mM NaCl, 2 mM MgCl_2_, 5% glycerol, 1 mM DTT, 0.01% NP-40), and bound proteins were eluted by TEV cleavage at 16°C for 2 h in the same buffer supplemented with 1 U/μL RiboLock. The eluate was applied to Flag-agarose beads (Anti-Flag M2 Affinity Gel, Sigma-Aldrich) for 10 h at 4°C, washed with 10 mL buffer, and eluted with Flag peptide. For cryo-EM, the final elution buffer contained 60 mM Tris-HCl, pH 8.0, 50 mM NaCl, 5 mM MgCl_2_, 2% glycerol, 0.01% NP-40, and 1 mM DTT.

#### Sucrose gradient centrifugation

Eluates from split-tag tandem affinity purifications were loaded onto linear 10–40% (w/v) sucrose gradients in 60 mM Tris-HCl (pH 8.0), 50 mM NaCl, 2 mM MgCl_2_, 0.003% NP-40, and 1 mM DTT, and centrifuged for 16 h at 129,300 × g, 4°C. Gradients were fractionated into 15 fractions, which were precipitated with 10% trichloroacetic acid. TCA-precipitated proteins were resuspended in sample buffer, separated by SDS-PAGE, and visualized by colloidal Coomassie staining (Roti-Blue, Roth).

#### Mass spectrometry

Prominent Coomassie-stained bands were excised and identified by MALDI-TOF mass spectrometry. Semi-quantitative mass spectrometry was performed at FingerPrints proteomics, University of Dundee, UK. MaxQuant software was used to analyze raw data.^53^

#### Growth analysis of Rrp12 mutants

To analyze cell growth, yeast cells were plated in 10-fold serial dilutions on YPD plates and incubated for 2 days at 23°C, 30°C, and 37°C.

#### Immunofluorescence

For siRNA transfection, 1.2 × 10ˆ5 293T cells were seeded into each well of 6-well plates. After 14 h, cells were transfected with 40 pmol siRNA using 7.5 μL Lipofectamine RNAiMAX (Thermo Fisher Scientific, 13778) per well, according to the manufacturer’s instructions. After 56 h of transfection, cells were detached with TrypLE (Gibco, 12604021) and reseeded into 35-mm confocal dishes pre-coated with 0.1 mg/mL poly-D-lysine (Beyotime, ST508), at a density of 2 × 10ˆ5 cells per dish. The remaining cells were seeded into 24-well plates for western blot analysis. After an additional 16 h, cells prepared for western blotting were lysed directly in 1x SDS loading buffer. Cells grown in confocal dishes were fixed with 4% paraformaldehyde (Adamas Life, F8011) for 15 min at room temperature, washed three times with PBS, and permeabilized with 0.1% Triton X-100 (Sangon Biotech, A110694-0100) in PBS for 8 min. After five washes with PBS, cells were blocked with blocking buffer containing 5% bovine serum albumin in PBS for 1 h at room temperature. Cells were then incubated with primary antibodies diluted in blocking buffer overnight at 4°C. After five washes with PBS, cells were incubated with secondary antibodies diluted in blocking buffer for 1 h at room temperature in the dark. Following another five washes with PBS, nuclei were stained with DAPI (Sigma, D9542) diluted 1:1000 in PBS for 5 min, followed by five additional PBS washes. Finally, 1 mL PBS was added to each dish to preserve cell morphology before imaging. Fluorescence images were acquired using a Leica TCS SP8 confocal microscope equipped with a 40x oil-immersion objective.

#### Electron microscopy and image processing

A 3.5 μL purified Enp1-Rrp12 WT or Enp1-Rrp12 ΔC samples were applied to Quantifoil R1.2/1.3 holey-carbon grids, pre-coated with 2 nm carbon. The grids were blotted for 4–5 s at 4°C and then plunge-frozen in liquid ethane using a FEI Vitrobot Mark IV. Data collection was performed on a Titan Krios cryo-electron microscope operating at 300 keV, using EPU2 software for automatic acquisition. Micrographs were recorded with a pixel size of 1.146 Å and 1.045 Å, respectively, and a defocus range of −1 to −2.5 μm, using a Falcon IV direct electron detector in EER format under low-dose conditions (total dose ∼44 e^–^/Å^2^ or ∼50 e^–^/Å^2^, respectively). Original image stacks were dose-weighted, aligned, summed, and drift-corrected using MotionCor2.^54^ Contrast-transfer function (CTF) parameters and resolutions were estimated for each micrograph using CTFFIND4.^55^ Micrographs with an estimated resolution of less than 5 Å and an astigmatism of less than 5% were manually screened for contamination or carbon rupture.

For particle picking, 18,637 and 10,675 good micrographs from the Rrp12 WT and Rrp12 ΔC datasets, respectively, were selected for further analysis. A total of 1,909,316 and 1,436,572 particles were automatically picked using Gautomatch v0.56, without a reference, from these two datasets.

For Rrp12 WT dataset, reference-free 2D classification was performed, yielding two main subsets: 90S pre-ribosome (541,416 particles) and pre-40S ribosome (383,764 particles), as shown in Figure S1. These subsets were then separately subjected to 3D classification in Relion v5.0.^61^ For 90S pre-ribosome subset, during the first round of alignment-free 3D classification, particles were sorted into four classes using a sphere mask automatically generated in Relion v5.0 (T-value = 4).^61^ Two classes displaying state A and state B1 features were selected for further refinement.

In the pre-40S ribosome subset, three classes were selected after the initial round of 3D classification for further analysis. A second round of focused 3D classification was then performed using sphere masks centered on Tsr1 or Utp14. From class 2, a total of 12,066, 16,473, and 13,035 particles were assigned to states Tsr1-1, Tsr1-2, and Tsr1-3, respectively. A total of 26,387 particles from class 3 and 52,758 particles from class 4 were identified as state Rrp12-A1. Additionally, 71,779 particles from class 2 were assigned to state Rrp12-A2, and 85,384 particles from class 4 corresponded to state pre-Rio2-C.

For Rrp12 ΔC dataset, a total of 1,436,572 particles were extracted using box size of 480 and 360 pixel in parallel. Then heterogeneous refinement was performed in cryoSPARC v4.5.3^56^ using either 90S pre-ribosome (EMD-10052) or pre-40S ribosome (EMD-4337) as references, respectively. Two classes from 480-box dataset were selected as 90S pre-ribosome subset, and 329,648 particles from 360-box dataset were identified as pre-40S ribosome subset.

These two subsets were re-imported into Relion v5.0^61^ for 3D classification. For 90S subset, two classes displaying state a or state B1 features were selected for further refinement. A second round of focused 3D classification with a spherical mask near the 3′ major domain further separated the state B1 particles into two sub-classes, termed state B1 and B1^∗^, respectively.

For the pre-40S subset, three classes were selected after the first 3D classification in Relion v5.0.^61^ The class1 and class3 then applied to the second round of 3D classification, while class 4 was subjected to focused classification using a spherical mask focused on Utp14. This results in four distinct classes: 211,537 particles from class 1 and 23,872 from class 3 were assigned to state Rrp12-A1^∗^; 6,707 particles from class 3 were assigned to state Tsr1-1^∗^; 29,832 particles from class 4 were assigned to state Rrp12-A1^∗∗^; and 3,017 particles from class 4 corresponded to state pre-Rio2-C^∗^.

Final reconstructions were obtained by re-extracting the selected particles in Relion at 1.146 Å or 1.045 Å/pixel followed by refinement in Relion v5.0.^61^ CTF refinement and multibody refinement were applied to improve map quality. For pre-40S particles, maps were divided into head and body regions for refinement. Post-processing, local resolution filtering, and sharpening were performed using Relion’s auto-generated masks and DeepEMhancer v0.13.^51^

#### Model building and refinement

Published *C. thermophilum* 90S structures (PDB: 6RXY, 6RXT, 6RXU)^11^ were used as initial models and rigid-body fitted into the cryo-EM density maps of states a, A, B1, and B1^∗^ using ChimeraX v1.8.^50^ Minor manual adjustments were subsequently performed in Coot^52^ to optimize model-to-map fit. Due to the limited resolution and the inability to unambiguously identify the associated snoRNP, no model was built for this additional density in the 90S state B1^∗^.

In general, for the pre-40S ribosome structures, the structures of yeast and human pre-ribosome (PDB: 7WTP, 7WTT, 6EML)^19^^,^^36^ were used as initial references for positioning assembly factors. Corresponding *C. thermophilum* models of the assembly factors were retrieved from the AlphaFold database^43^ and aligned to their yeast or human homologs. These were then assembled manually in Coot v0.9.5^52^ to generate the initial models, and adjust manually to fit into their corresponding density map.

In detail, for states Tsr1, the *S. cerevisiae* model (PDB code: 7WTP)^19^ was used as the initial reference. The uS2/uS5/eS21 cluster was fitted using AlphaFold models,^43^ and for Tsr1-2 and Tsr1-3 states, Tsr1 was modeled using AlphaFold prediction^43^ with rigid-body fitting in Coot v0.9.5.^52^ The unresolved regions of Tsr1 protein (state Tsr1-2: aa.1-56, 343–470; state Tsr1-3: aa.1-56, 348–470) were manually removed in Coot.^52^

For states Rrp12, the human RRP12-A1 structure (PDB: 7WTT)^19^ served as a reference, and manual fitting was performed in Coot.^52^ Rrp12 in this state does not have sufficient resolution, thus all the side chains were removed. For the pre-Rio2-C state, the yeast model (PDB: 6EML)^36^ was used as a reference.

For specific states from the Rrp12 ΔC sample, including Tsr1-1^∗^, Rrp12-A1^∗^, Rrp12-A1^∗∗^, AlphaFold models^43^ of Rio1 and Utp14 were fitted to the density and truncated where unresolved regions were absent. Except in Rrp12-A1^∗^, h44 of the 18S rRNA was excluded from the final model due to lack of corresponding density in other pre-40S states. Due to the low resolution of the density map, the final model of the Tsr1-1^∗^ state was only subjected to rigid-body fitting in Coot without further refinement; therefore, no refinement statistics are provided. Similarly, for the pre-Rio2-C^∗^ state, no molecular model was built.

The final models were real-space refined with secondary structure restraints using the PHENIX suite v1.19.^58^ Final model evaluation was performed with MolProbity.^62^ Maps and models were visualized and figures were created with ChimeraX v1.8.^50^

### Quantification and statistical analysis

MaxQuant^53^ software was used to analyze semiquantitative mass spectrometry data, according to the user manual documentation. Normalized data is given in Table S1. Details of the cryo-EM analysis is described in the method details and Tables S2 and S3. No statistical analysis was used in this study.

### Additional resources

There are no additional resources in this study.

## Supplemental information

Document S1. Figures S1–S7 and Tables S2 and S3

Table S1. Source data of the label-free quantitative LC-MS/MS, related to Figure 1

Document S2. Article plus supplemental information
