# Supplementary material for: Nucleoplasmic checkpoint of the 40S ribosomal decoding center maturation
Source: Cell Rep. 2026 Jun 11;45(6):117545. doi: 10.1016/j.celrep.2026.117545 (PMC13291541; doi:10.1016/j.celrep.2026.117545)
Supplement: Document S1. Figures S1–S7 and Tables S2 and S3 [file mmc1.pdf]

Cell Reports, Volume *45*

Supplemental information

Nucleoplasmic checkpoint of the 40S ribosomal decoding center maturation

Benjamin Lau, Yi Li, Jingyi Zhu, Xianwen Ye, Paulina Fischer, Xiaying Hong, Rui Yuan, Roland Beckmann, Ed Hurt, and Jingdong Cheng

SUPPLEMENTAL FIGURES AND FIGURE LEGENDS

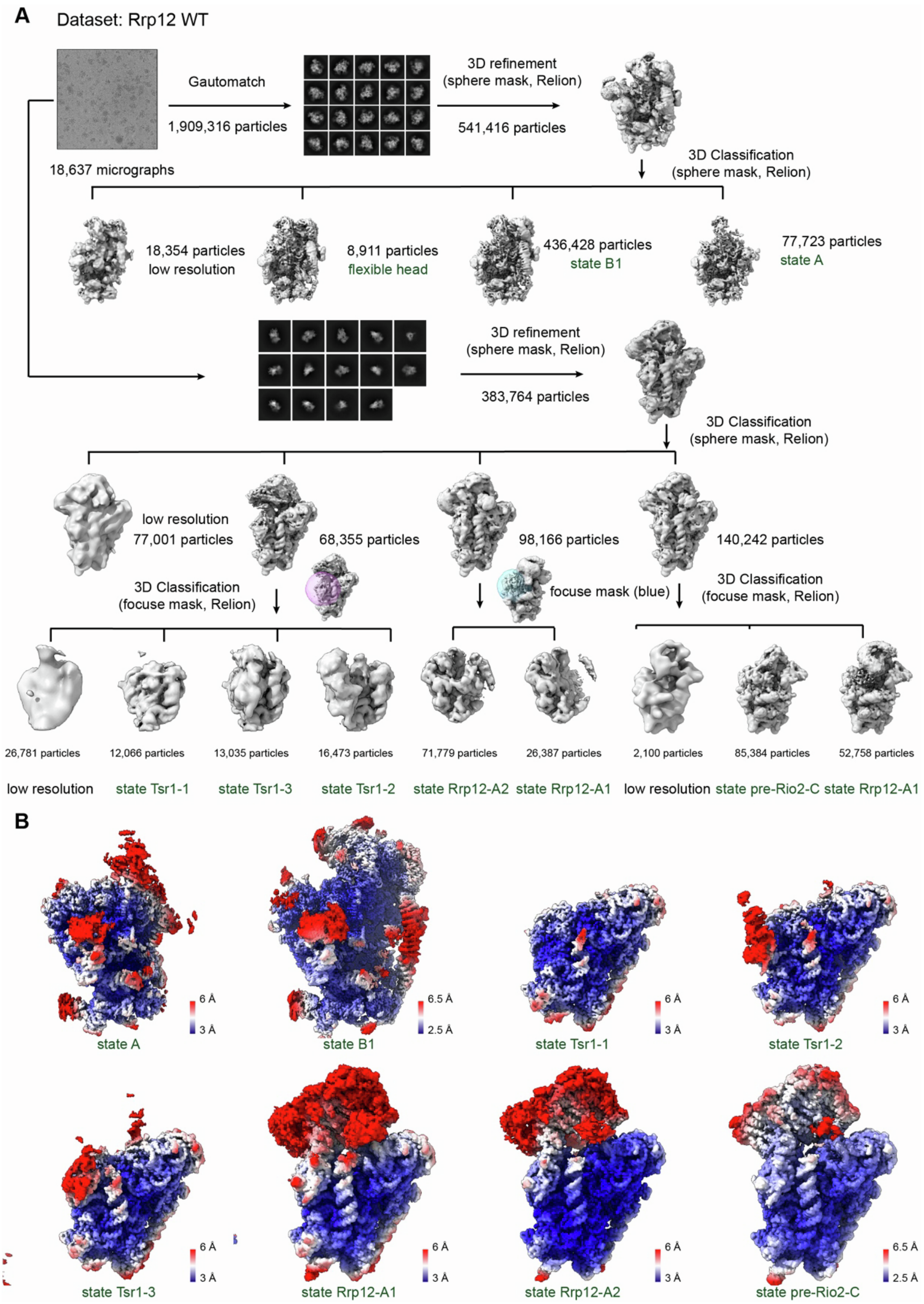

Figure S1. Cryo-EM data processing of the Rrp12 WT sample, Related to Figure 2

(A) Cryo-EM data processing workflow for Rrp12 WT dataset. The masks, software and the key parameters used during various steps of data processing are indicated. Two classes of 90S particles and six classes of pre-40S particles were selected for final refinement.

(B) Local-resolution distributions of the final cryo-EM reconstructions for the eight resolved states, estimated by Relion and indicated by a blue-to-red color scale.

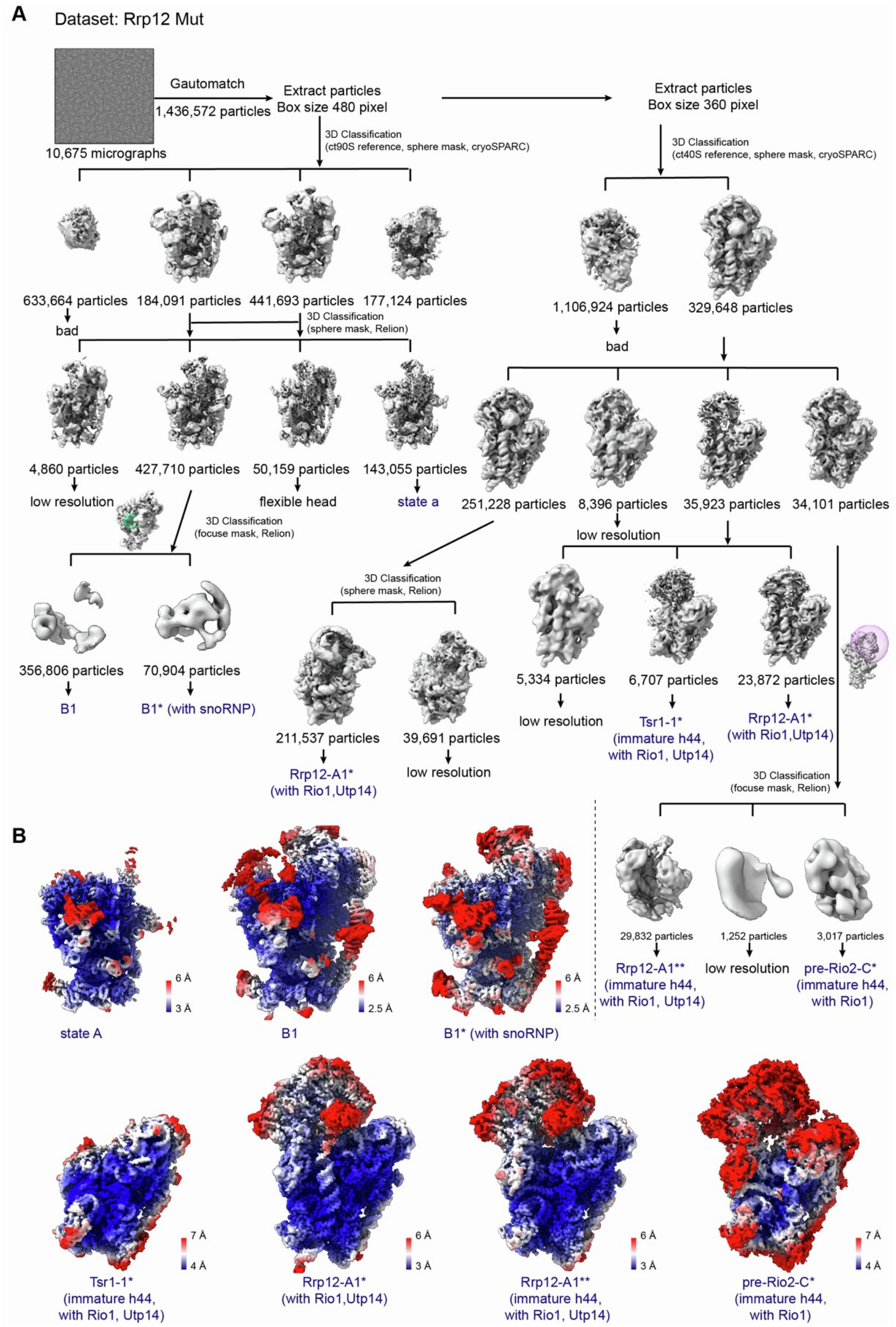

Figure S2. Cryo-EM data processing of the Rrp12 ΔC sample, Related to Figure 3

(A) Cryo-EM data processing workflow for Rrp12 ΔC dataset. The masks, software and the key parameters used during various steps of data processing are indicated. Three classes of 90S particles and four classes of pre-40S particles were selected for final refinement.

(**B**) Local-resolution distributions of the final cryo-EM reconstructions for the seven resolved states, estimated by Relion and indicated by a blue-to-red color scale.

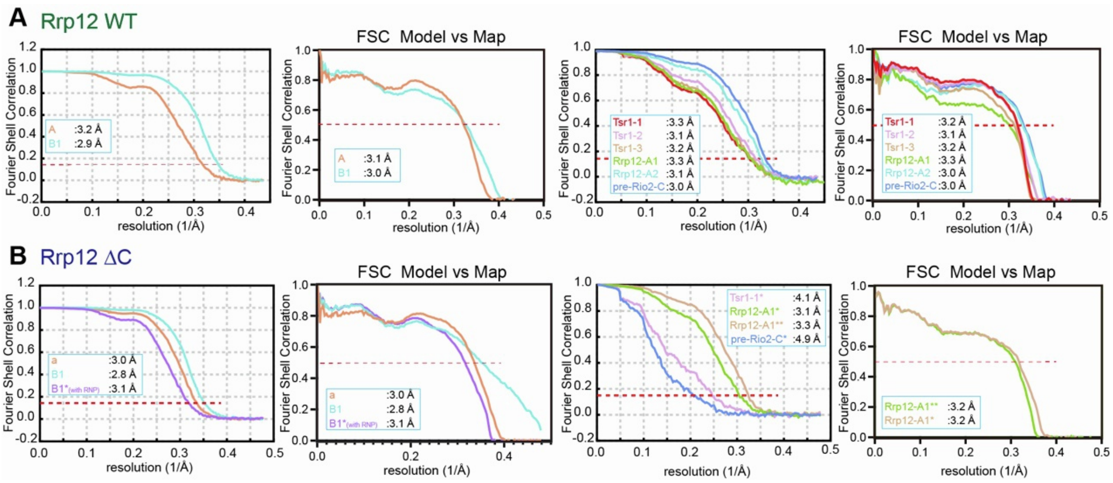

Figure S3. Cryo-EM structural analysis of Rrp12 WT and Rrp12 ΔC maps, Related to Figure 2 and 3

(A and B) Fourier shell correlation (FSC) curves for cryo-EM maps of Enp1-Rrp12 WT sample (A) and Enp1-Rrp12 ΔC sample (B) particles. For each dataset, FSC curves between two half-maps (left panels) and between the refined model and the final map (right panels) are shown. Individual FSC curves are shown for each resolved structural state, as indicated in the legend.

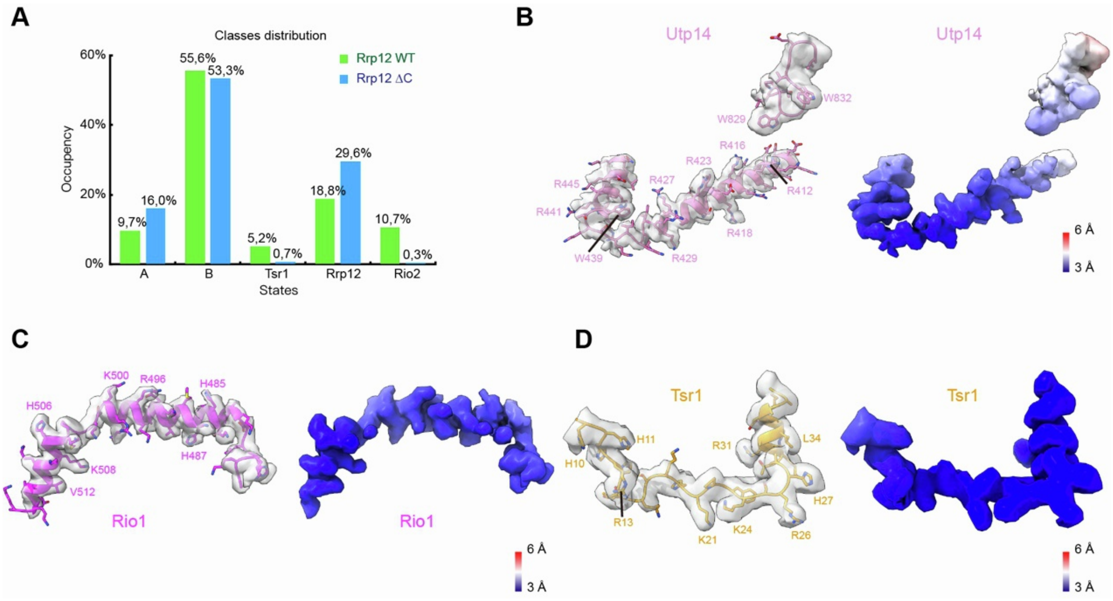

Figure S4. Particle distribution and structural details of Utp14, Rio1 and Tsr1, Related to Figures 2-6

(A) Proportional distribution of particle populations across distinct assembly states in Rrp12 WT and Rrp12 ΔC samples.

(B-D) Close-up views of the cryo-EM densities for Utp14 (aa. 403-451, 825-837), Rio1 (aa. 475-517) in state Rrp12-A1** and Tsr1 (aa. 10-38) in state Rrp12-A1, the corresponding local resolution was shown on right.

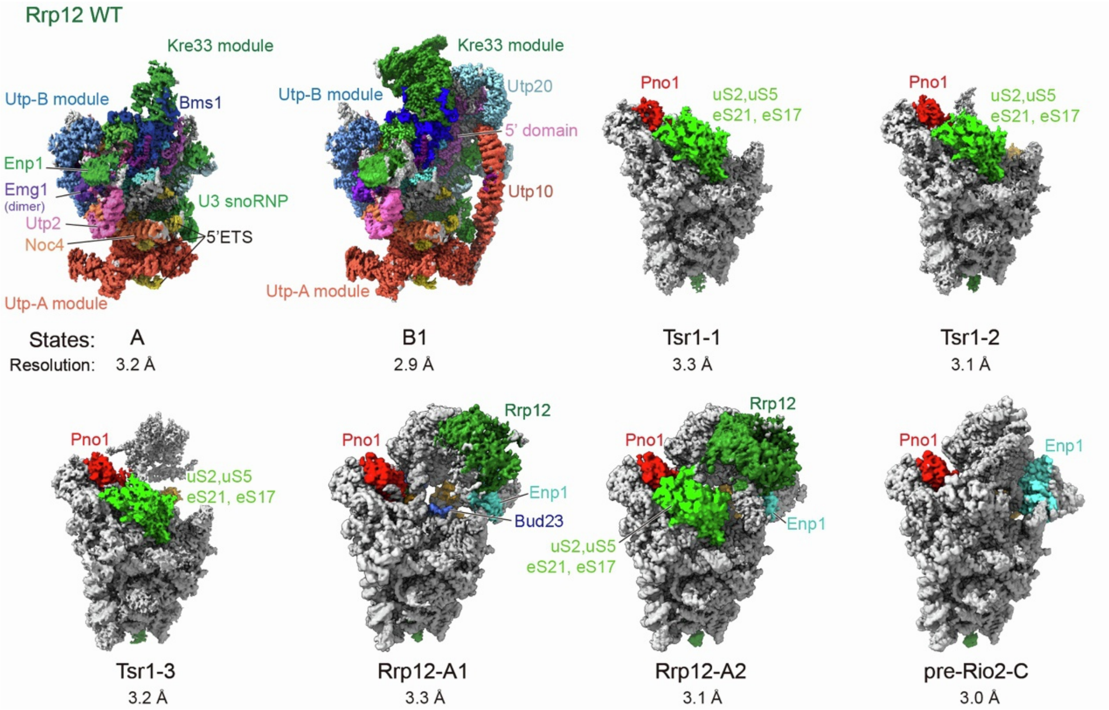

Figure S5. Cryo-EM reconstructions of Enp1-Rrp12 WT particles, Related to Figure 2

Rotated views of the density maps in Figure 2.

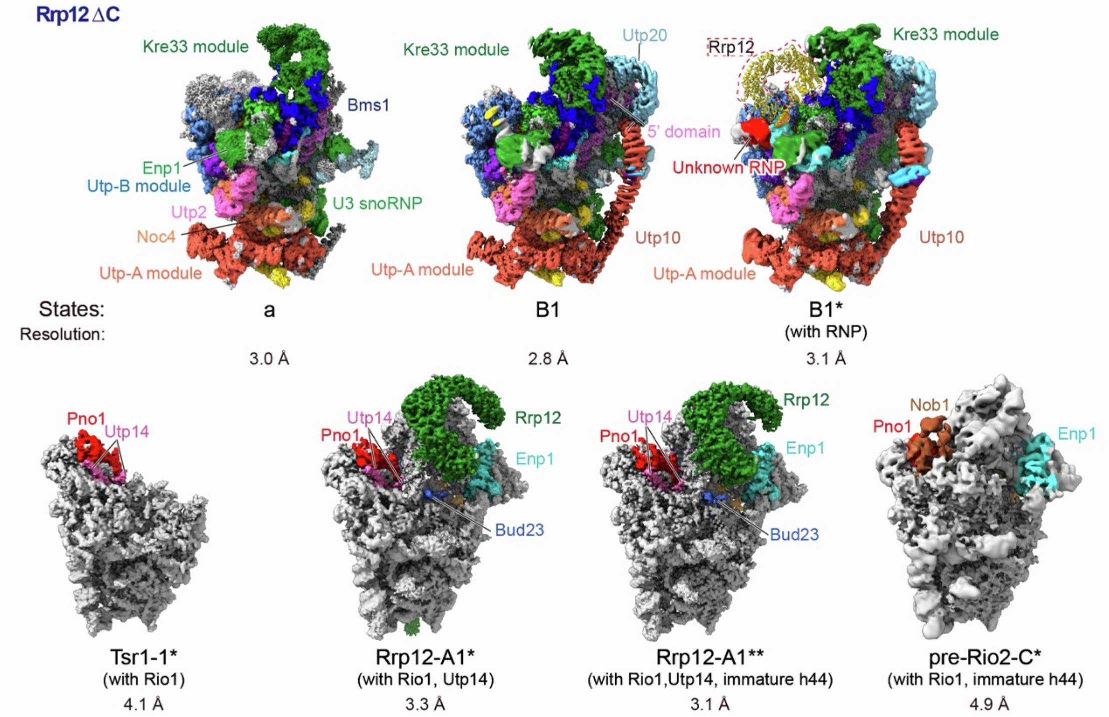

Figure S6. Cryo-EM reconstructions of Enp1-Rrp12 ΔC particles, Related to Figure 3

Rotated views of the density maps in Figure 3.

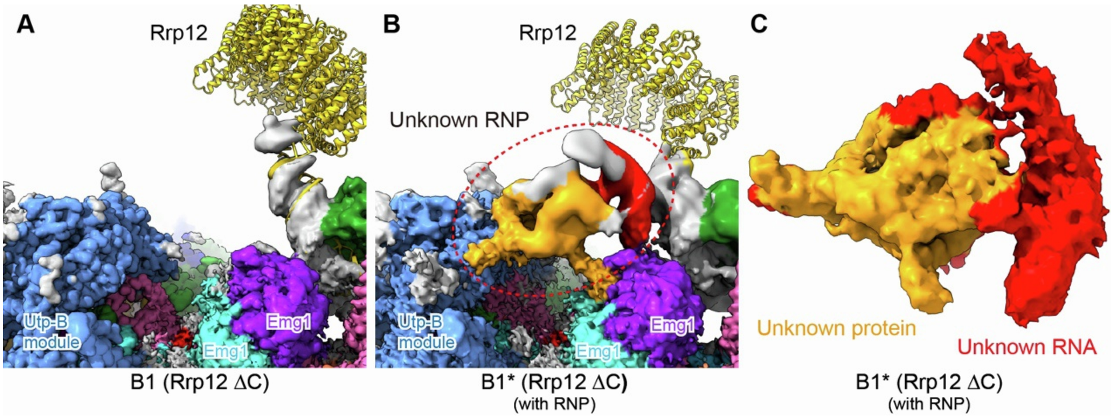

Figure S7. Putative snoRNP associated with Rrp12 near the 3' major domain, Related to Figure 3

(A and B) Structure comparison between states B1 and B1* show an extra unknown density in B1* display features consistent with both protein(orange) and RNA(red).

(C) Close-up views of the cryo-EM densities for the putative snoRNP.

Table S2. Cryo-EM data collection, refinement and validation statistics (Rrp12 WT sample), Related to Figure 2

	A	B1	Tsr1-1	Tsr1-2	Tsr1-3	Rrp12-A1	Rrp12-A2	Pre-Rio2-C
**Data collection and processing**								
Magnification	105,000	105,000	105,000	105,000	105,000	105,000	105,000	105,000
Voltage (kV)	300	300	300	300	300	300	300	300
Electron exposure (e–/Å^2)	~50	~50	~50	~50	~50	~50	~50	~50
Defocus range (μm)	-1 to -2.5	-1 to -2.5	-1 to -2.5	-1 to -2.5	-1 to -2.5	-1 to -2.5	-1 to -2.5	-1 to -2.5
Pixel size (Å)	1.146	1.146	1.146	1.146	1.146	1.146	1.146	1.146
Symmetry imposed	*C1*	*C1*	*C1*	*C1*	*C1*	*C1*	*C1*	*C1*
Initial particle images (no.)	1909316	1909316	1909316	1909316	1909316	1909316	1909316	1909316
Final particle images (no.)	77723	436428	12066	16473	13035	52758	71779	85384
Map resolution (Å)	3.2	2.9	3.3	3.1	3.2	3.3	3.1	3.0
FSC threshold	0.143	0.143	0.143	0.143	0.143	0.143	0.143	0.143
Map resolution range (Å)	2.8-18	2.5-10	2.9-20	2.9-14	2.9-14	2.9-20	2.6-16	2.7-10
**Refinement**								
Initial model used (PDB code)	6RXT	6RXU	N/A	N/A	N/A	N/A	N/A	N/A
Model resolution (Å)	3.1	3.0	3.2	3.1	3.2	3.3	3.0	3.0
FSC threshold	0.5	0.5	0.5	0.5	0.5	0.5	0.5	0.5
Map sharpening *B* factor (Å^2)	-81	-92	-68	-61	-67	-65	-75	-82
Model composition								
Non-hydrogen atoms	179899	227977	50717	55764	55608	80162	84775	76911
Protein residues	19273	24305	2967	3601	3586	5862	6453	5065
RNA	1419	1817	1274	1274	1272	1582	1582	1720
Ligands	4	4	3	3	3	3	3	3
B factors (Å^2)	46.01	23.96	41.42	40.13	28.35	25.48	56.64	53.83
Protein	38.69	21.70	26.51	37.38	24.93	25.31	40.08	30.44
RNA	82.02	35.01	54.33	43.03	31.93	25.71	81.67	79.48
Ligand	130.67	24.32	38.58	41.16	28.54	24.18	53.51	43.57
R.m.s. deviations								
Bond lengths (Å)	0.003	0.006	0.004	0.003	0.003	0.004	0.006	0.004
Bond angles (°)	0.760	0.865	0.786	0.750	0.746	0.775	0.858	0.828
Validation								
MolProbity score	1.50	1.59	1.57	1.58	1.59	1.59	1.55	1.57
Clashscore	4.86	5.40	4.91	4.96	5.32	5.68	5.08	5.22
Poor rotamers (%)	0.02	0.03	0.16	0.07	0.00	0.12	0.09	0.26
Ramachandran plot								
Favored (%)	96.29	95.75	95.53	95.42	95.65	95.97	95.97	95.79
Allowed (%)	3.67	4.17	4.44	4.55	4.29	3.97	3.97	4.17
Disallowed (%)	0.04	0.08	0.03	0.03	0.06	0.05	0.06	0.04
**EMDB**	**66677**	**66678**	**66679**	**66680**	**66681**	**66682**	**66683**	**66684**
**PDB**	**9XA7**	**9XA8**	**9XA9**	**9XAA**	**9XAB**	**9XAC**	**9XAD**	**9XAE**

Table S3. Cryo-EM data collection, refinement and validation statistics (Rrp12 ΔC sample), Related to Figure 3

	a	B1	B1*(with snoRNP)	Tsr1-1*	Rrp12-A1*	Rrp12-A1**	Pre-Rio2-C*
**Data collection and processing**							
Magnification	130,000	130,000	130,000	130,000	130,000	130,000	130,000
Voltage (kV)	300	300	300	300	300	300	300
Electron exposure (e–/Å^2)	~44	~44	~44	~44	~44	~44	~44
Defocus range (μm)	-1 to -2.5	-1 to -2.5	-1 to -2.5	-1 to -2.5	-1 to -2.5	-1 to -2.5	-1 to -2.5
Pixel size (Å)	1.045	1.045	1.045	1.045	1.045	1.045	1.045
Symmetry imposed	*C1*	*C1*	*C1*	*C1*	*C1*	*C1*	*C1*
Initial particle images (no.)	1436572	1436572	1436572	1436572	1436572	1436572	1436572
Final particle images (no.)	143055	356806	70904	6707	211537	29832	3017
Map resolution (Å)	3.0	2.8	3.1	4.1	3.1	3.3	4.9
FSC threshold	0.143	0.143	0.143	0.143	0.143	0.143	0.143
Map resolution range (Å)	2.5-14	2.1-12	2.8-12	3.6-22	2.8-14	2.9-16	3.9-18
**Refinement**							
Initial model used (PDB code)	6RXT	6RXU	N/A	N/A	N/A	N/A	N/A
Model resolution (Å)	3.0	2.8	3.1		3.2	3.2	
FSC threshold	0.5	0.5	0.5		0.5	0.5	
Map sharpening *B* factor (Å^2)	-74	-80	-70	-61	-67	-60	-90
Model composition							
Non-hydrogen	177230	227688	228232		81055	78440	
atoms	18941	24305	24374		5966	5966	
Protein residues	1419	1804	1804		1584	1462	
RNA	4	4	4		4	4	
Ligands							
B factors (Å^2)	52.36	21.08	86.33		20.55	28.92	
Protein	41.03	19.05	82.81		20.21	29.30	
RNA	107.25	31.05	103.67		21.04	28.35	
Ligand	109.71	20.74	90.88		21.12	30.70	
R.m.s. deviations							
Bond lengths (Å)	0.004	0.005	0.006		0.005	0.007	
Bond angles (°)	0.810	0.890	0.999		0.841	0.949	
Validation							
MolProbity score	1.53	1.55	1.70		1.62	1.69	
Clashscore	5.10	5.19	6.77		5.99	6.49	
Poor rotamers (%)	0.04	0.02	0.11		0.10	0.24	
Ramachandran plot							
Favored (%)	96.17	95.98	95.33		95.82	95.28	
Allowed (%)	3.78	3.96	4.55		4.14	4.65	
Disallowed (%)	0.05	0.06	0.12		0.03	0.07	
**EMDB**	**66685**	**66686**	**66687**	**66688**	**66689**	**66690**	**66691**
**PDB**	**9XAF**	**9XAG**	**9XAH**		**9XAJ**	**9XAK**	
